# Systematic review on the biology, ecology, genetic diversity and parasite transmission potential of *Panstrongylus geniculatus* (Latreille 1811) in Latin America

**DOI:** 10.1590/0074-02760200528

**Published:** 2021-02-26

**Authors:** Ricardo José Vivas, Jorge Enrique García, Felipe Guhl, Carolina Hernández, Natalia Velásquez, Juan David Ramírez, Julio César Carranza, Gustavo Adolfo Vallejo

**Affiliations:** 1 Universidad del Tolima, Laboratorio de Investigaciones en Parasitología Tropical, Ibagué, Colombia; 2 Universidad de Ibagué, Facultad de Ciencias Naturales y Matemáticas, Ibagué, Colombia; 3 Universidad de los Andes, Centro de Investigaciones en Microbiología y Parasitología Tropical, Bogotá, Colombia; 4 Universidad del Rosario, Facultad de Ciencias Naturales y Matemáticas, Departamento de Biología, Grupo de Investigaciones Microbiológicas, Bogotá, Colombia

**Keywords:** Panstrongylus geniculatus, geographic distribution, genetic diversity, oral transmission of *Trypanosoma cruzi*, climate change

## Abstract

*Panstrongylus geniculatus* (Latreille, 1811) is the triatomine with the largest geographic distribution in Latin America. It has been reported in 18 countries from southern Mexico to northern Argentina, including the Caribbean islands. Although most reports indicate that *P. geniculatus* has wild habitats, this species has intrusive habits regarding human dwellings mainly located in intermediate deforested areas. It is attracted by artificial light from urban and rural buildings, raising the risk of transmission of *Trypanosoma cruzi*. Despite the wide body of published information on *P. geniculatus*, many knowledge gaps exist about its biology and epidemiological potential. For this reason, we analysed the literature for *P. geniculatus* in Scopus, PubMed, Scielo, Google Scholar and the BibTriv3.0 databases to update existing knowledge and provide better information on its geographic distribution, life cycle, genetic diversity, evidence of intrusion and domiciliation, vector-related circulating discrete taxonomic units, possible role in oral *T. cruzi* transmission, and the effect of climate change on its biology and epidemiology.

Chagas disease affects around 7 million people in Latin America and is considered one of the 10 most important neglected diseases in the region.^1^ ^,^ ^2^ Currently, 154 species within the Triatominae subfamily (three extinct and 151 extant) have been described and are able to transmit *Trypanosoma cruzi*, the causative agent of Chagas disease under natural and experimental conditions.^2^ ^,^ ^3^ ^,^ ^4^ ^,^ ^5^ ^,^ ^6^ ^,^ ^7^ ^,^ ^8^

*Panstrongylus geniculatus* (Latreille, 1811) is the triatomine with the greatest geographic distribution in the Americas, ranging from southern Mexico to northern Argentina and including the Caribbean islands.^9^ ^,^ ^10^ This vector occupies extremely variable areas across the wild landscape, being found in different ecosystems within the same country.^11^ Most records deal with the species occupying altitudes ranging from sea level to around 2,000 metres above sea level (masl); however, the species has been found at altitudes from 2,000 to 4,000 masl in some Andean countries (i.e., Colombia, Venezuela, Ecuador, Peru and Bolivia), thereby indicating the species’ great adaptive capability, probably enabling its shift in altitude as a consequence of lowland warming, regarding today’s climate changes.^12^ ^,^ ^13^ ^,^ ^14^ ^,^ ^15^ ^,^ ^16^

The last few decades have seen much interest in the biology, ecology and epidemiology of *P. geniculatus*, thereby enabling effective control strategies for this species to be designed. Therefore, this review aims to update existing knowledge about *P. geniculatus* and address current information gaps. A search was conducted in Scopus, PubMed, Scielo, Google Scholar, BibTriv3.0 databases with no filters of language or time and until August 2020. All relevant studies on taxonomy, morphological variability, life cycle, geographical distribution, genetic diversity, intrusion and colonisation of human dwellings, oral transmission and discrete taxonomic units of *T. cruzi* and possible effects of climate change on *P. geniculatus* were chosen.

Taxonomy, morphology and life cycle

*Taxonomy* - Currently, *P. geniculatus* (Latreille, 1811) is the accepted scientific name for this species; however, the following synonyms have also been used reviewed by Patterson et al.:^17^ *Reduvius geniculatus* (Latreille, 1811), *Conorhinus lutulentus* (Erichson, 1848), *Lamus geniculatus* (Stal, 1859), *Conorhinus corticalis* (Walker, 1873), *Conorhinus geniculatus* (Walker, 1873), *Lamus corticalis* (Lethierry & Severin, 1896), *Triatoma geniculata* (Chagas, 1912), *Triatoma tenuis* (Neiva, 1914), *Triatoma fluminensi*s (Neiva and Pinto, 1922), *Mestor geniculatus* (Brindley, 1931) and *Panstrongylus parageniculatu*s (Ortiz, 1971).

Fifteen species have been described in the *Panstrongylus* genus: *P. chinai*, *P. diasi*, *P. geniculatus*, *P. guentheri*, *P. howardi*, *P. hispaniolae*, *P. humeralis*, *P. lenti*, *P. lignarius*, *P. lutzi*, *P. megistus*, *P. martinezorum*, *P. mitarakaensis*, *P. rufotuberculatus*, *P. tupynambai*.^2^ ^,^ ^3^ ^,^ ^17^ Studies on the taxonomy of this genus and the identification of *P. geniculatus* have been problematic because most are based on morphometry and karyotyping, but few have employed molecular markers.^17^ ^,^ ^18^ ^,^ ^19^ ^,^ ^20^ ^)^ The phylogenies based on morphological traits presents the *Panstrongylus* genus as a monophyletic group, whereas the ITS-2 (rDNA) phylogenetic trees suggest that it is polyphyletic group of *Triatoma* species from South, Central and North America.^9^ ^,^ ^21^ In another study, using four mitochondrial markers (16S, COI, COII, *Cytb*) and two nuclear (18S and 28S) ones, *Panstrongylus* fell into two groups. One of the groups contained *P. tupynambai*, *P. lutzi* and *P. geniculatus* as sister taxa of *Nesotriatoma*, whereas the other was a highly supported group that includes *Triatoma tibiamaculata* and *P. megistus*.^22^

The use of morphological characters to classify *Panstrongylus* species has led to the description of ‘new species’ in the genus. This is the case for two species described in French Guiana and Venezuela; namely, *P. mitarakaensis* and *P. martinezorum*, respectively.^23^ ^,^ ^24^ They both look quite similar to *P. geniculatus* but no molecular methods have been used to confirm that they are the same species.^3^ Another species in Venezuela, *P. turpiali*, was synonymised with *P. chinai* but both were then shown to phenotypically match *P. geniculatus*.^25^ ^,^ ^26^ ^,^ ^27^ Therefore, it is necessary to use molecular approaches to clarify the taxonomic status of *P. geniculatus* and phenotypically similar species.

*Morphological variability in P. geniculatus* - Several authors have reported on the morphological characteristics of *P. geniculatus*. Its nymph and adult stages have been described using light and scanning electron microscopy.^9^ ^,^ ^17^ ^,^ ^19^ ^,^ ^28^ ^,^ ^29^ ^,^ ^30^ ^,^ ^31^ ^,^ ^32^ ^,^ ^33^ ^,^ ^34^ ^,^ ^35^ ^,^ ^36^ Elsewhere, the geometric morphometric techniques applied to a sylvatic population and its laboratory descendants over five generations revealed size differences in *P. geniculatus* but not in its shape. Essentially, head size and wing size were both reduced from sylvatic and laboratory populations.^35^ Another morphometric study on *P. geniculatus* showed a reduction in the size of its populations in Caracas city, when compared with sylvatic populations from other Venezuelan regions.^20^ In this review we have included unpublished photographs representing the known morphological characteristics of the species, which we believe will help to a primary identification of individuals captured in sylvatic, peridomestic or domestic environments (Fig. 1A-G).

**Fig. 1:**
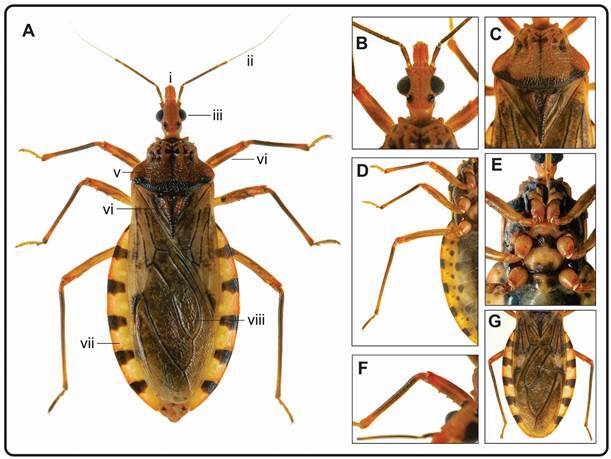
(A) dorsal view of a female *Panstrongylus geniculatus*: (i) clipeus, (ii) antenna, (iii) compound eye, (iv) leg, (v) pronotum, (vi) scutellum, (vii) connexivum, (viii) wings (hemielytrons); (B) head; (C) pronotum and scutellum; (D) legs; (E) coxa and trochanter; (F) spicules; (G) connexivum. Source: authors.

Adult *P. geniculatus* insects display sexual dimorphism. The total length of the male is 22-28 mm and the larger-bodied female is 22.5-29.5 mm. The posterior end of the male’s abdomen is rounded in shape, whereas it is tip-shaped in the female (Fig. 2I-J). Its colouration is light brown to orange brown and is interspersed with dark patches on various parts of the body, which do not follow a defined pattern in that the connexivum has two intercalated regions of colour: a flat black-coloured one and another coloured orange to ochre (yellow-orange) in the interior (Fig. 1A).^36^

The head is sub-conical-shaped, narrow at eye level, and shorter than the pronotum; the anteocular region is approximately twice as long as the postocular region. The dorsal surface is slightly rough. The general colouration is ochre, but in some individuals two dark stripes extending from the base of the jugae to the space between the ocelli are observed in the dorsal region. Anteniferous tubers are located near the anterior edges of the eyes, and the antennas vary from reddish brown to black. The first segment of the antenna subtly exceeds the apex level of the clypeus (Fig. 1A-B).

Behind the head is the orange-brown pronotum whose anterior lobe is slightly convex and rough. The pronotum features a central four-leaf clover-shaped mark and “1 + 1” side marks. The posterior lobe is irregularly rough, with a black band along the posterior margin, except in the humeral area (Fig. 1C). The femurs and tibiae are dark brown or black (Fig. 1A, D). The legs have light yellow or orange-yellow coxas and trochanters (Fig. 1E). The front and middle femurs have two to six (usually four) small subapical denticles arranged in two rows (Fig. 1F). The hemielytrons are light brown or light yellowish brown, with dark veins in the membranes (Fig. 1G).

*Life cycle of P. geniculatus under laboratory conditions* - *P. geniculatus* eggs do not adhere to the substrate; rather, they are oviposited free on a surface. They are oblong and symmetrically shaped, without lateral flattening or collar, and at one end is a prominent and convex operculum through which hatching is performed. The eggs are bright white for the first days after oviposition, and six-eight days later they turn pinkish yellow, pink, or pearl, becoming translucent after hatching (Fig. 2A-C). The nymphal and adult stages are shown in Fig. 2D-J.

**Fig. 2:**
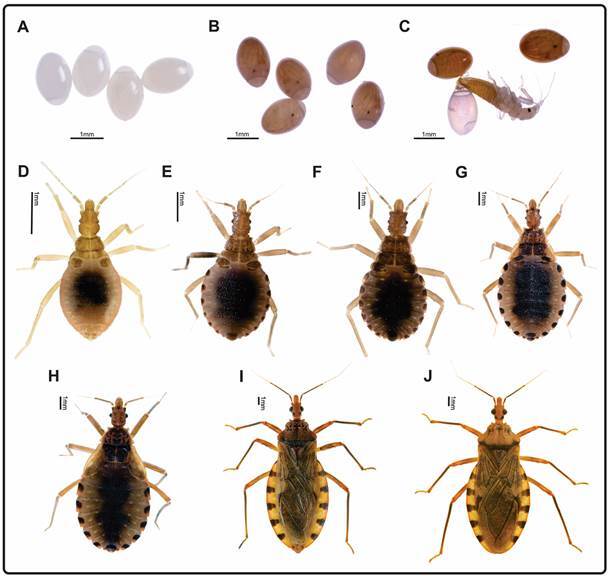
stages of the life cycle of *Panstrongylus geniculatus*: (A) freshly oviposited eggs; (B) eggs near hatching; (C) hatching; (D) nymph stage N1; (E) nymph stage N2; (F) nymph stage N3; (G) nymph stage N4; (H) nymph stage N5; (I) adult (female); (J) adult (male). Source: authors.

The life cycle period under laboratory conditions has been shown to be dependent on the experimental conditions employed, such as temperature, relative humidity and food source in the colonies. Depending on the management of these variables, very heterogeneous records have been observed from 149.5 to 531 days, and even up to two years. When the experimental conditions are 21-26ºC and 90-100% for temperature and relative humidity, respectively, life cycle durations of 269, 297, 275.8, 273, 275.4 days have been reported (average, 278 days).^32^ ^,^ ^37^ ^,^ ^38^ ^,^ ^39^ ^,^ ^40^ ^,^ ^41^ ^,^ ^42^ ^,^ ^43^ ^,^ ^44^ One of the first studies to investigate the life cycle of *P. geniculatus* reported that the total development time is approximately 2 years.^45^ Another study reported on the difficulties of establishing the species as a colony and observed that the time taken for the life cycle is 531 days (75.8 weeks).^37^

The 1990s brought advances in biological knowledge. The life cycle was studied under controlled laboratory conditions and reported that the average development time was 269 days (38.4 weeks) and 297 days (42.4 weeks) for nymphs fed on chickens (*Gallus gallus*) and opossums (*Didelphis marsupialis*), respectively.^38^ In a life-cycle study using uncontrolled conditions, the insects were subjected to temperatures of 21-25ºC, relative humidities of 90-100%, and were fed *ad libitum* weekly on chickens (*G. gallus*). An N1 nymph stage to adulthood duration of 275.8 days (39.4 weeks) was reported.^39^ The duration of the life cycle for specimens from the municipality of Amalfi in Antioquia, Colombia, which were subject to controlled temperature (29 ± 1ºC) and relative humidity (95 ± 1ºC) conditions, was 149.5 days (21.4 weeks).^32^ Later on, evidence of the colonisation processes in Amalfi in Antioquia, Colombia, and estimated transmission risk indicator values were obtained. They also reported on the time required for blood intake by adults (mean, 67.8 minutes for males and 80.27 minutes for females). Blood consumption was on average 0.12 g for males and 0.26 g for females, with defecation times post-feeding of 55 minutes for females and 60 minutes for males.^40^ Sylvatic intrusive insects captured in domestic environments have had high *T. cruzi* infection prevalence (see Table III in this review). In addition, a post-feeding time close to one hour for both males and females could be a determining factor for this species regarding the oral transmission of Chagas’ disease due to food contamination. As *P. geniculatus* has been repeatedly associated with orally transmitted outbreaks, this hypothesis could be ascertained by comparing this species’ post-feeding time characteristics and natural infection frequencies to those for other species invading human dwellings.

The first statistical evaluation of the population dynamics of *P. geniculatus* under controlled laboratory conditions studied a cohort of 60 eggs placed at constant temperature (26 ± 3ºC) and relative humidity (90 ± 10%) that were fed every 15 days on chickens (*G. gallus*). The researchers observed that the hatching percentage was 88.9%, the average nymph development time was 273 days (39 weeks), and the longevity of the adults was 504 days (72 weeks).^41^

In contrast, the need of optimising the ovipositors of female *P. geniculatus* to enable massive breeding of this species in the laboratory and for use in bioassays, compared the ovipositors of laboratory and field populations fed on chickens (*G. gallus*) and mice (*Mus musculus*, strain BALB/c). The authors found that the fertility of the first-generation laboratory population was twice that of the wild population when both were fed on chickens.^42^ ^,^ ^43^

The most recent and detailed study on the vital statistics of *P. geniculatus*, estimated its development times by stages, vital statistics (mortality and fertility), and population growth parameters (intrinsic natural growth rate, finite population growth rate, net reproduction rate, and generational time). In a cohort of 100 eggs kept under constant temperature (26 ± 1ºC) and relative humidity (60 ± 10%) and fed every eight days with chicken blood, it was observed that the total development time by stage was 275.4 days (39.4 weeks), and mortality reached 58% in the initial stages (I, II, and III), which represents 60% of the total mortality.^44^

Geographical distribution, ecotopes and genetic variability

*Geographical distribution and ecotopes* - *P. geniculatus* has the greatest geographical distribution of species belonging to this genus. It is found exclusively in Latin America between 21.1ºN, 30.3ºS, 38.8ºE, and 94.9ºW, covering an estimated 12 million km^2^. To date, its presence has been reported in 18 countries (Table I, Fig. 3), from southern Mexico to northern Argentina, including the Caribbean islands.^9^ ^,^ ^12^ ^,^ ^45^ ^,^ ^46^ ^,^ ^47^

**TABLE I:** Geographic distribution of *Panstrongylus geniculatus* in Latin America

Country	Location	Reference
Argentina	Chaco, Corrientes, Entre Rios, Formosa, Misiones, Santa Fe and Santiago Del Estero	9,10
Bolivia	Beni, Cochabamba, La Paz, Santa Cruz and Tarija	9,10,48,49,50,51
Brazil	Acre, Amapá, Amazonas, Bahia, Brasília DF, Ceará, Espírito Santo, Goiás, Maranhão, Mato Grosso, Minas Gerais, Pará, Paraná, Piauí, Rio de Janeiro, Rondônia, Roraima, São Paulo and Tocantins	9,10, 46, 52,53,54,55,56
Colombia	Amazonas, Antioquia, Arauca, Atlantico, Bolivar, Boyaca, Caqueta, Casanare, Cauca, Cesar, Choco, Cordoba, Cundinamarca, Guainia, Guaviare, Huila, La Guajira, Magdalena, Meta, Norte de Santander, Putumayo, Risaralda, Santander, Tolima, Sucre, Valle del Cauca, Vaupes and Vichada	9,10,15,57,58,59,60,61,62,63
Costa Rica	Alajuela, Cartago, Guanacaste, Heredia, Limon, Puntarenas and San Jose	9,11,64
Ecuador	Esmeraldas, Imbabura, Manabi, Napo, Orellana, Pastaza, Pichincha and Sucumbios	9,10,65,66,67,68,69
Guatemala	ND	9
British Guiana	ND	9
French Guiana	Apotou, Awala-Yalimapo, Cayenne, Comopi, Grand-Santi, Kourou, Iracoubo, Macouria, Mana, Maripasoula, Matoury, Montsinery, Ouanry, Papaichton, Roura, Remire-Montjoly, Regina, Saint-Elie, Saint-Georges, Saint-Laurent, Sinnamary and Saul	9,70,71
Mexico	Chiapas, Veracruz and Yucatan	10
Nicaragua	Boaco and Zelaya	9,72,73
Panama	Panama	9,74
Paraguay	Alto Parana, Boqueron, Concepcion, Caaguazu, Nueva Asuncion and Paraguari	9,10,46
Peru	Amazonas, Ayacucho, Bagua, Cajamarca, Cerro de Pasco, Cusco, Huanuco, Jaen, Junin, Loreto, Madre de Dios, Martin, Puno, San Ignacio, San Pasca, Ucayali and Utcubama	9,16,46,75,76,77
Suriname	Commewijne, Para, Paramaribo, Saramacca, Sipaliwini and Wanica	9,78,79
Trinidad y Tobago	San Patricio and ST. George	9,80
Uruguay	ND	9
Venezuela	Amazonas, Anzoategui, Aragua, Bolivar, Carabobo, Distrito federal, Delta Amacuro, Falcon, Guarico, Lara, Merida, Miranda, Monagas, Tachira, Yaracuy and Zulia	9,10,81

**Fig. 3:**
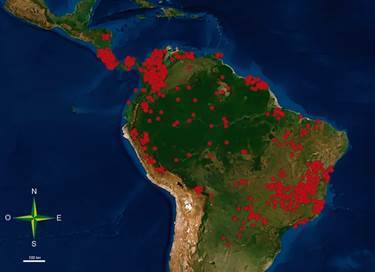
continental distribution of *Panstrongylus geniculatus*. Maps were made with the database reported by Ceccarelli et al.^47^

With its wide geographical distribution, *P. geniculatus* is found from sea level to 2,000-4,000 m above sea level in the Andean countries (Colombia, Venezuela, Ecuador, Peru and Bolivia)^14^ ^,^ ^15^ ^,^ ^16^ and in places where the average annual temperature (minimum and maximum) ranges between -3.9 and 34.3ºC and precipitation reaches up to 4,000 mm/year.^9^ Some authors have suggested that the morphological variations found among *P. geniculatus* populations result from the demands of the different environments.^18^ Table II shows the presence of *P. geniculatus* above 2,000 masl in Colombia and Peru.

**TABLE II:** Records of *Panstrongylus geniculatus* presence above 2,000 masl in Colombia and Peru

Country	Locality	State	Coordinates	masl	Reference
Colombia	Boavita	Boyaca	6.333333 N -72.666667 W	2,350	14,15
	Susacon	Boyaca	6.2298 N -72.6901 W	2,480	
	San Mateo	Boyaca	6.3303 N -72.585 W	2,200	
	Casabianca	Tolima	5.0796 N -75.1206	2,100	
Peru	Ninacaca	Pasco	-10.75819 S -76.17819 W	4,100	14,16
	Tarma	Junin	-11.419335 S -75.68849 W	3,050	
	Cholon	Huanuco	-8.690057 S -76.733652 W	2,350	
	Cutervo	Cajamarca	-6.376045 S -78.821275 W	2,600	
	San Miguel	Ayacucho	-12.932081 S -73.747786 W	2,660	
	Huanta	Ayacucho	-12.938411 S -74.250212 W	2,630	

masl: metres above sea level.

Natural ecotopes vary from dry or very dry tropical forests to savanna and humid tropical forests, and *P. geniculatus* can be found in caverns, caves, and burrows in these environments where it is mainly associated with armadillos (Dasypodidae), anteaters (Myrmecophagidae), bats (Chiroptera), and a wide variety of other vertebrates. It is also found under bark, tree trunks and fallen trees and near bird nests in palm trees (*Acrocomia aculeata*, *Syagrus romanzoffiana*, *Elaeis oleifera*, and *Leopoldinia piassaba*) and in epiphytes.^9^ ^,^ ^17^ ^,^ ^82^ ^,^ ^83^ ^,^ ^84^ ^,^ ^85^ Interestingly, it was reported that the species displays entomophagic behavior towards moths (*Eacles* spp.) in the forests of French Guyana for the first time, which confirms its versatility for survival in environments with low food availability.^86^

Although the presence of *P. geniculatus* has been recorded in 18 countries, the recent availability of predictive maps can produce a robust summary of its distribution and show its spatial variation so that regions with a high probable risk of vector transmission and transmission through food contamination can be identified.^87^

*Genetic diversity in P. geniculatus* - The *Panstrongylus* genus is classified as belonging to the Triatomini, a monophyletic tribe. However, the phylogenetic relationships inside this group are far to be cleared because current knowledge is limited and messy.^3^ The Triatomini tribe is divided into three lineages: *T. dispar*, North American, and South American lineages. *Panstrongylus* is included in the Antillean Triatoma + Panstrongylus clade within the North American lineage. Three groups were tentatively distinguished in the *Panstrongylus* subclade, but only one of them includes *P. geniculatus* and its closest relatives *P. mitarakaensis*, *P. martinezorum*;^3^ it also includes a set of south Amazonian species as *P. lutzi* (and its synonym *P. sherlocki*), *P. lenti*, *P. diasi*, *P. guentheri* and *P. tupynambai*.^9^ ^,^ ^17^ ^,^ ^23^ ^,^ ^24^ ^,^ ^88^ ^,^ ^89^

Regarding karyotype variations in *Panstrongylus* species, it has been established that the species (*P. chinai*, *P. geniculatus*, *P. lignarius*, *P. rufotuberculatus*, *P. tupynambai*, *P. herreri*) have 20 autosomes (except *P. megistus* that has 18 autosomes), and X_1_X_2_Y chromosomes for males and X_1_X_1_X_2_X_2_ for females. In a study, three clusters were defined based on the C-band pattern and meiotic chromosomal behavior where *P. geniculatus* was not located in any of the groupings because it shares characteristics with each group. Consequently, the authors proposed that *P. geniculatus* should be considered as a complex of species comprising at least two different species because of its high chromosomal variability, wide distribution, and high phenotypic variation;^18^ molecular evidence, however, is needed to confirm this hypothesis.

A Venezuelan study conducted a genetic variability analysis on *P. geniculatus* using *Cytb* as the molecular marker because it is extensively used to determine genetic polymorphisms in triatomines. The results showed that this marker is useful for describing genetic heterogeneity in *P. geniculatus* and revealed spatial and genetic patterns that agree with domiciliation processes for this species, but it could not resolve the evolutionary relationships. Even though this marker is informative about the population structure, other genes must be evaluated to study the genetic variability.^90^

A very recent study used DNA markers and molecular data to study genetic diversity in *P. geniculatus* with the aim obtaining information about the processes that have shaped genetic diversity in this species.^91^ Using mitochondrial DNA (mtDNA) fragments, 16S rRNA and *Cytb*, the authors reconstructed a phylogenetic tree that showed that *P. geniculatus* from Colombia and Venezuela form a monophyletic species with four clades concordant with its geographic distribution, which is partly explained by the Andes uplift (Fig. 4). However, other factors, including anthropogenic and eco-epidemiological effects must also be investigated to explain the existence of recent geographic *P. geniculatus* lineages. The authors also reconstructed the relationships within the *Panstrongylus* genus using various genetic markers. Using mtDNA markers (16S, ND4 and *Cytb*), they detected two clades: one includes *P. lutzi* and *P. tupynambai*, which is the sister clade of *P. geniculatus* (geniculatus group), and the other one includes *P. lignarius* and *P. megistus*, which represent the sister clade of *P. rufotuberculatus*, *P. howardi* and *P. chinai* (megistus group).

**Fig. 4:**
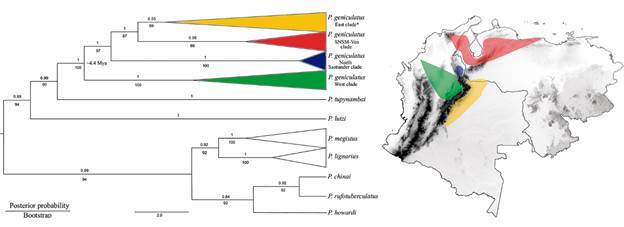
genetic differentiation of *Panstrongylus geniculatus* in Colombia and Venezuela reported by Caicedo-Garzón et al.^91^

To conclude, further studies using more genetic markers and genomic approaches, along with widespread sampling from different geographic locations are needed to unravel the inter- and intra-specific relationships of the *Panstrongylus* genus, and specially for *P. geniculatus* considering its domiciliation reports, high infection rates with *T. cruzi* and incrimination in oral outbreaks of Chagas disease in Colombia and Venezuela. All this knowledge is relevant for the development of control and surveillance strategies for Chagas disease vectors.

**Epidemiology, intrusion, colonisation of human habits and possible role in the oral transmission of *T. cruzi***

*Evidence of dwelling intrusion by P. geniculatus in Latin America* - Although *P. geniculatus* is considered a species of wild habits, several cases of intrusion into homes have been reported in different Latin American countries in recent decades. This new scenario implies an increased risk of transmission in endemic and non-endemic areas of Chagas disease, either by direct contact or via food contamination.^92^ ^,^ ^93^ Table III shows 45 registered reports of intrusion by adult *P. geniculatus* in Bolivia, Brazil, Colombia, Peru, Surinam and Venezuela. Despite growing awareness of the relevance of the transmission dynamics of *P. geniculatus*, the drivers of house invasion remain poorly understood. However, it was observed that invasion by this species decreased with higher landscape disturbance levels and in hotter-day municipalities and increased somewhat in more places with intermediate disturbance, peaking in municipalities with average rainfall.^94^

**TABLE III:** *Panstrongylus geniculatus* adult specimens reported inside human dwellings in Latin American countries, without evidence of colonization (eggs, nymphs and exuviae)

Country	Department/State	*Trypanosoma cruzi* infection frequency	Reference
Argentina	Corrientes	ND	95
Bolivia	La Paz	33%	49
	Cochabamba	62,5%	51
Brazil	Paraná	Negative	96
	Piauí	Positive	54
	Goiás	1,40%	97
	Mato Grosso do Sul	3,20%	98
	Rio de Janeiro	Negative	99,100
	Amazonas	Positive	81,101
	Amazonas	Negative	102,103
	Minas Gerais	ND-Negative	104,105,106
	Distrito Federal	Negative	107
	Goiás	7,70%	108
	São Paulo	Negative	109,110
	Acre	Negative	111
Colombia	Bolivar	ND	112
	Meta	70,6-100%	93,113
	Magdalena	83%	114
	Casanare	ND	115
	Cordoba	30-50%	92,116
	Santander	56%	117
	Sucre	58,82%	118
Peru	Amazonas, Cajamarca	ND	76
	Pasco	18,20-50%	119,120
	Cusco	ND	121
	Loreto	ND	122
	San Martin	ND	123
Surinam	Saramacca, Wanica	ND	124
Venezuela	Caracas	38,86-53%	125,126
	Merida	Positive	127
	Caracas, Merida, Vargas	76,1% (average)	128
	Lara	ND	129
	Aragua	Negative	130
	Aragua, Caracas, Miranda, Vargas	50,3% (average of the four states)	84
	Sucre	ND	131

ND: no data.

It is accepted that the increased interactions occurring between triatomines and humans are mainly related to losses in vegetation cover and increases in urban and rural populations invading natural ecotopes. Most of these intrusions occur at night when *P. geniculatus* fly in from wild environments to inhabited areas under the attraction of artificial lighting in search of food.^84^ The author concluded that this occurred regardless of the type of housing, and that the invasion increased when high numbers of domestic animals were present and the distance to the forest was small.^84^ Recently, 11 *P. geniculatus* specimens were captured in the city of Bucaramanga, a Colombian neighborhood, of which five out of nine tested were positive for *T. cruzi* (56%), which drew attention to the risk of infection by this vector settling in the periphery of homes and adjacent to the natural ecotopes where its life cycle in the wild occurs.^117^

The ability of *P. geniculatus* to fly up to 2 km away has been verified.^125^ In other study, was reported that *P. geniculatus* is attracted to artificial light sources. These researchers installed a light trap positioned 45 m above sea level in a wild area of Manaus in the Amazonas State of Brazil, and they operated it monthly for three consecutive nights over the course of a year. They found that the most commonly captured triatomine was *P. geniculatus* (38 individuals) in addition to six other species.^132^ Likewise, Jacome et al., determined the risk associated with the dispersive nocturnal flights of sylvatic triatomines by installing artificial lights in a model house in the northeastern plains of Colombia and concluded that this nocturnal dynamic poses a risk for the domestic introduction of discrete typing units (DTUs) of *T. cruzi* associated with sylvatic transmission foci.^133^

**Evidence of intradomiciliary and peridomiciliary colonisation of *P. geniculatus* in Latin America**

Although *P. geniculatus* is registered as an intrusive wild species in some regions of the Americas, in other regions it is registered as a species capable of colonising human dwellings. These differences in the behavior of *P. geniculatus* probably reflect the genetic differences in this species across Latin American populations. Generally, the reported number of *P. geniculatus* nymphs associated with human dwellings varies, and Table IV lists nine publications that have reported on the presence of *P. geniculatus* eggs and nymphs. Furthermore, two intradomiciled adults and three peridomiciled nymphs in Muñecas, La Paz (Bolivia) were recorded.^48^

**TABLE IV:** Registration of *Panstrongylus geniculatus* adults, nymphs and eggs inside houses or peridomiciles in Latin American countries

Country	Department/State	ID	PD	Association with	Natural intection	Reference
Bolivia	La Paz	X	X	ND	Negative	48
Brazil	Pará	X	X	Pigs	17%	134
Colombia	Antioquia	X	ND	ND	21,2-50%	40
	Atlantico	X	ND	ND	ND	135
	Casanare	X	ND	ND	Negative	136
Venezuela	Miranda	X	ND	Rats	20%	137
	Lara	X	ND	ND	9,0-5,07%	138,139
	Metropolitan District of Caracas	X	X	ND	59,5%	140

ND: no data; ID: intradomicily; PD: peridomicily.

Fifteen pigsties were examined in Muana, Pará State, Brazil, contained 207 females, 168 males, 2 N5, 7 NIII, 16 NII, 28 NI stages and 385 unhatched eggs, thereby evidencing a domiciliation process for *P. geniculatus*. In 10 of the 15 houses located up to a 10 m distant from the pigsties, 21 adults were collected in total.^134^

The presence of *P. geniculatus* adults in 80 homes in Amalfi, Antioquia, Colombia, but only in one of the homes did they find nymphal stages of the insect was recorded.^40^ Thirty-seven adults and a nymph of *P. geniculatus* in the intra-domicile of the studied dwellings in Atlántico, Colombia were reported.^135^ The presence of adults and nymphs in development stages IV and V of *P. geniculatus* in the intra-domiciles in six of seven villages in the municipality of Tamara in Casanare Department (Colombia) was recorded.^136^

Two males and one female *P. geniculatus*, 5NV, 8NIV, 3NIII, and 1NII stages, and seven eggs were captured in the tunnel of a rat (*Rattus rattus*) inside a house in Miranda, Venezuela. All the triatomines were abundantly blood-fed, with the exception of one recently molted male and one female, whose emergences were confirmed by the presence of NV-stage exuviae. Fecal examination of the three NV-stages and an adult male revealed the presence of *T. cruzi*.^137^ In three houses examined in El Guamito, Lara State, Venezuela, were found one female, six N5-, two NIII- and two N2-stages and an overall *T. cruzi* infection rate of 9%.^138^ In another study in the same state, *P. geniculatus* was found in 11 homes, registering 136 adults and two NIII stages, with a colonisation index of 18.18% and a 5.07% infection rate for *T. cruzi*.^139^ 3390 adult *P. geniculatus* specimens, 26 NV, 71 NIV and 27 NIII stages were captured in the Metropolitan District of Caracas between 2007 and 2013, of which 59,5% carried *T. cruzi*. The authors warned that dramatic modifications to the surrounding natural habitats had led to the establishment of a *T. cruzi* urban enzootic cycle, resulting in a high risk of transmission of Chagas disease in this capital city.^140^

*T. cruzi* isolates from *P. geniculatu*s, *R. rattus* and from humans infected during an orally-acquired Chagas disease outbreak in a non-endemic region of Venezuela belong to a same *T. cruzi* I genotype (TcId). The similarity in the parasite isolates from the three patients affected by the urban oral outbreak, the triatomine, and the rat captured at the guava juice preparation site allowed the authors to suggest that the source of infection was common among these persons and the vector, providing indirect evidence of *P. geniculatus* domiciliation in this region.^141^

The colonisation capacity of *P. geniculatus* has been studied under laboratory conditions. One such study reported a ro/b index of 0.77, but concluded that as a “K” strategist with a low reproduction rate and a prolonged generational time, it was unlikely that *P. geniculatus* was an important disease vector at the household level, a finding that does not presently reflect the epidemiological importance of the species.^41^ Nevertheless, another study compared the results they obtained from *P. geniculatus* (ro/b = 0.74) with two species known for their high colonising capacity, *Rhodnius prolixus* (0.74) and *Triatoma infestans* (0.65), and concluded that *P. geniculatus* has a remarkable colonising capacity, which should alert the relevant institutions to the risk presented by this species and the need to design new control strategies aimed at the wild species that in recent decades have been increasingly reported as having home intrusion and colonisation behavior.^44^

The presence of sexual dimorphism was an indicator of home adaptation and the authors concluded that populations of *P. geniculatus* from Caracas, Venezuela, are morphologically adapted to home environments.^20^ That the ability of the species to adapt to different environments in a few generations as was recently confirmed by Nakad-Bechara et al.^90^ and poses a future risk to humans and should alert health institutions, especially taking into account the possible new scenarios of climate change and the specific anthropic processes of deforestation and landscape fragmentation.

***P. geniculatus* and oral transmission of *T. cruzi***

Over the last two decades, the oral form of disease transmission has changed from being a rare and unusual event to a public health concern after causing numerous deaths.^142^ ^,^ ^143^

The difficulty in obtaining direct evidence incriminating the vector species responsible for the oral transmission of *T. cruzi*, is related to the fact that oral Chagas disease outbreaks occur several days after food contamination. Despite this difficulty, in some reported cases strong suspicions exist about the role of *P. geniculatus* in this transmission mechanism, because it has been observed as an abundant species in the area of the outbreak and it has been found invading the houses where contaminated food was prepared and, although in some cases the captured insects did not carry *T. cruzi* parasites, in other cases high levels of natural infection were observed. Table V lists 11 publications that report indirect evidence involving *P. geniculatus* in the oral transmission of *T. cruzi*.

**TABLE V:** Cases where *Panstrongylus geniculatus* has been reported as a highly probable transmitter of oral *Trypanosoma cruzi* infection in Latin America

Country	Location	Contaminated food	Cases/deaths	Reference
	Restrepo (Meta)	Arepa and/or pineapple juice	4/0	113
	Antioquia (Turbo)	ND	11/1	144
	Santander (Bucaramanga)	tangerine juice and/or orange juice	9/0	143
Colombia	Santander (Lebrija)	tangerine juice and/or orange juice	10/2	145
	Santander (Piedecuesta)	ND	5/0	146
	San Vicente de Chucuri	ND	3/0	143
	Cesar (Aguachica)	ND	11/0	147
Bolivia	Beni (Guayaramerin)	majo juice	14/0	148
Venezuela	Caracas	guava juice	103/1	149
	Vargas	ND	85/4	150
	Merida (Antonio Pinto Salinas)	ND	5/0	151

ND: no data.

Venezuela has experienced the most numerous and acute cases of parasite transmission by the oral route, with the source of infection generally being the intake of juices contaminated with triatomine feces. Infections have been reported in greater Caracas and the states of Falcon, Merida, Tachira, and Vargas where *P. geniculatus* has been the species linked with most of the outbreaks. Because it is the most abundant species in these localities, it has high *T. cruzi* infection rates and has been observed to defecate while returning to its shelter after feeding ^128^ ^,^ ^140^ ^,^ ^152^

In the municipality of Chacao in Caracas in 2007, 103 people from a school were infected with *T. cruzi*, and of those infected, 19% needed hospitalisation and one child died. The infection source was juice made in a house where *T. cruzi*-infected *P. geniculatus* insects were captured.^149^ During 2009, another outbreak occurred in a school environment that affected 82 people, from which three children and one adult died. Again, the evidence from the case indicates that the infection source was juice contaminated with the feces of *P. geniculatus*.^150^

In Colombia, several cases of infection by oral transmission have occurred in areas with low endemicity and presence of domiciled vectors. Thus, wild vectors such as *P. geniculatus* have become important in relation to this mechanism of infection and may be associated with outbreaks related to oral transmission in several Colombian municipalities.^153^ In Lebrija, Santander, an acute case was presented, where ten infections and two deaths were reported.^145^ A report on a new scenario for transmission in the department of Córdoba-Colombia in an area that was not considered to be a region at risk of *T. cruzi* transmission. This report was based on an acute case of the disease in which the patient did not present lesions on the skin or in the periocular region that indicated the bite of the insect.^92^

The transmission sources for two oral outbreaks were identified, one of them in the Colombian town of Restrepo, Meta. By epidemiological analysis, genotyping and allele identification of seven *T. cruzi* microsatellites in the samples obtained from patients, as well as insects and reservoirs implicated in the outbreaks, they determined that this outbreak was caused by fecal contamination of arepa and pineapple juices by *P. geniculatus*. Their distance analysis allowed them to observe the grouping of patients with insects rather than with the reservoirs. Their findings were further explained by recent housing construction in the outbreak area, which altered the natural habitat of *P. geniculatus* and caused its intrusion into homes.^113^

**DTUs of *T. cruzi* detected in *P. geniculatus***

Natural infections of *T. cruzi* in *P. geniculatus* have been widely reported and quantified in different Latin American countries. Despite no clear trend, high rates of natural infection occur with this parasite, reaching 44% on average. Brazilian rates are 16.5%,^154^ 50%,^155^ 4.7%^156^ and 27.3%.^157^ The Trinidadian rate is 42.5%,^80^ the Colombian rates are 50%,^40^ 70.6%^93^ and 58.3%,^118^ the Venezuelan rates are 20%^138^ and 76.1%,^128^ the Peruvian rate is 50%,^120^ and the Bolivian rate is 62.5%.^51^

Currently, seven DTUs, TcI to TcVI and T.c.bat, are known to circulate in the vectors in different proportions.^158^ ^,^ ^159^ The *T. cruzi* DTUs in *P. geniculatus* correspond to six of the seven described DTUs (TcI, TcII, TcIII, TcIV, TcV and TcVI), the geographic distributions of which in America for *P. geniculatus* are shown in Fig. 5. In Bolivia, insects infected with TcI and TcIII have been reported inside homes.^51^ In Brazil, insects infected with TcII and TcIII have been identified.^157^ In Colombia, the transmission dynamics of the primary and secondary vectors in six departments (Antioquia, Cesar, Guajira, Huila, Meta, and Norte de Santander) were studied and, with six circulating DTUs (TcI, TcII, TcIII, TcIV, TcV and TcVI). *P. geniculatus* was observed to be the species with the highest number of DTUs.^93^ ^,^ ^160^ While TcI, TcIII and TcIV genotypes were isolated from the insects collected in the Colombian department of Casanare,^133^ insects from Peru, Ecuador and French Guyana were only infected with TcI.^120^ ^,^ ^161^ Last, TcI, TcIII and TcIV DTUs were identified in *P. geniculatus* collected from Venezuela.^140^

**Fig. 5:**
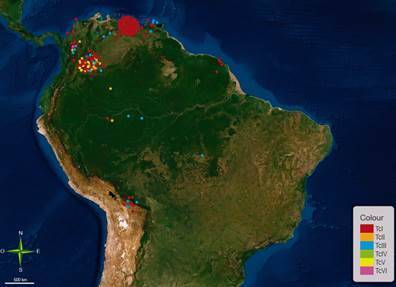
geographical distribution in America of *Trypanosoma cruzi* discrete typing units (DTUs) detected in *Panstrongylus geniculatus*.

**Possible effects of climate change on *P. geniculatus***

Although controversy persists about the consequences of climate change on vector-transmitted infectious diseases, there is full consensus that the global temperatures are closely linked with the physiological processes of arthropod vectors.^162^ ^,^ ^163^ Indeed, it has been shown that temperature and relative humidity can affect a vector insect by changing its original geographical distribution, altering the duration of its life cycle, or modifying its vector capacity, all which have consequences for the prevalence of infectious diseases.^164^

To date, several studies have aimed to establish the effect of environmental variables on triatomines; however, attention has been mainly directed to *T. infestans* and *R. prolixus*.^165^ ^,^ ^166^ ^,^ ^167^ No studies on *P. geniculatus* that assess the effect of climatic conditions on its life cycle or vector capacity have been reported under controlled laboratory conditions in the available databases. Only estimates of its potential distribution based on ecological niche modeling have been reported for some Latin American countries.

In Brazil, the geographic distribution of triatomine species in the Central West region was estimated and the climatic factors that influence their occurrence were analysed. The results indicate that among the variables analysed, temperature seasonality better explains the models of occurrence. The authors also reported that almost the entire region has the climatic conditions appropriate for at least one species and, according to the distribution maps, *P. geniculatus* was the most widespread species with the greatest potential for occurrence.^168^ In Venezuela, the possible effects of climate change on the potential distribution of five species (*Eratyrus mucronatus*, *P. geniculatus*, *T. maculata*, *R. robustus*, and *R. prolixus*) were analysed. It was found that the variables best explaining the model were seasonal temperature (47.9%) and isometry (26.5%), and the highest relative vector competence values were those for *R. prolixus* (0.8) and *P. geniculatus* (0.1). However, they concluded that the possible future effects of climate change on the vulnerability of the Venezuelan *P. geniculatus* population show a slight downwards trend.^169^

In Colombia, the geographical distribution of four species (*P. geniculatus*, *R. pallescens*, *R. prolixus*, and *T. maculata*) was estimated and identified a relationship between landscape structure and climate factors that influence their occurrence. According to the predictive maps on potential species distribution, the variables that best explain the model were altitude (26%) and precipitation seasonality (18%).^13^ In light of the potential future geographical distributions, expressed as the suitability of a climate niche, several papers agree that *P. geniculatus* has a wide geographic distribution and has a greater distribution potential in possible climate change scenarios.^13^ ^,^ ^168^ ^,^ ^169^

We recently observed that when *P. geniculatus* colonies are kept at 30ºC, they have higher mortality and lower fertility than those kept at 26 and 28ºC (Unpublished observations). Thus, it seems possible that under natural conditions, this species probably moves to areas where temperatures and altitudes favor its survival.

In Conclusion

All 15 of the species in the *Panstrongylus* genus have been described using morphological characters. Some of them are phenotypically similar to *P. geniculatus*, but molecular methods have not yet been used to determine whether or not they are the same species.

Geometric morphometry studies have revealed a reduction in the size of the populations studied in the urban areas of Caracas (Venezuela), when compared with sylvatic populations. The size reduction has also been verified in laboratory-raised populations, when compared with sylvatic populations; hence, this size reduction is likely associated with domiciliation processes.

Life-cycle timing under laboratory conditions has been shown to be dependent on the experimental conditions employed, such as temperature, relative humidity and the food source for the colonies. Depending on the management of these variables, very heterogeneous data have been recorded from 149.5-531 days to even up to two years. Other studies using temperature conditions of 21-26ºC and relative humidity of 90-100% have reported life cycle durations of 269, 297, 275.8, 273 and 275.4 days (average, 278 days).

Although *P. geniculatus* has been found in 18 countries, the recently generated predictive maps can produce a robust summary of its distributions. They can map its spatial variation to identify regions where a high risk of vector transmission of *T. cruzi* and transmission through food contamination are likely.

Based on the karyotype characteristics of *P. geniculatus*, which has high chromosomal variability*, P. geniculatus* is proposed to be a complex of species containing at least two different ones. The use of *Cytb* as a molecular marker has revealed spatial and genetic patterns related to domiciliation processes. The phylogenetic reconstruction of *P. geniculatus* from Colombia and Venezuela, using 16S rRNA and *Cytb*, showed that it comprises four phylogenetic clades concordant with its geographical distribution and the Andes uplift. However, it will be necessary to use other molecular markers and genomic approaches to identify intraspecific variations related to domiciliation of this species, high infection rates with *T. cruzi* and incrimination in oral outbreaks of Chagas disease in Colombia and Venezuela.

*P. geniculatus* is considered to have sylvatic habits; however, in recent decades at least 45 cases of intrusion into homes by adult insects have been reported in Bolivia, Brazil, Colombia, Peru, Surinam and Venezuela. This new scenario implies an increased transmission risk of the disease in endemic and non-endemic areas, either by direct contact or by food contamination. Nevertheless, in some Latin American regions the presence of nymphs, eggs and exuviae is evidence of colonisation of human dwellings, as reported in nine publications.^(40,48,134,135,136, 137,138,139,140)^ These differences in species behavior probably reflect genetic differences between the populations.

The difficulty in obtaining direct evidence incriminating which vector species is responsible for oral transmission of *T. cruzi*, is related to the fact that orally-transmitted outbreaks of Chagas disease occur several days after food contamination. Despite this difficulty, in some reported cases there are strong suspicions about the role of *P. geniculatus* in this transmission mechanism, because it has been abundantly observed in outbreak areas. Indeed, it has been found invading houses where contaminated food is prepared and although in some cases the captured insects did not show infection with *T. cruzi*, in other cases high levels of natural infection were observed. At least 10 publications present indirect evidence involving *P. geniculatus* in the oral transmission of *T. cruzi*.^113^ ^,^ ^143^ ^,^ ^144^ ^,^ ^145^ ^,^ ^146^ ^,^ ^147^ ^,^ ^148^ ^,^ ^149^ ^,^ ^150^ ^,^ ^151^

Although it is not completely clear what the effect of climate change will be on the biology of *P. geniculatus*, it is known that the species has optimal development between 26 and 28ºC and between 90-100% relative humidity. That colonies kept at 30ºC have high mortality and low fertility means that increases in temperature may affect the distribution of the species such that it could move altitudinally to find places with more favorable temperatures.
